# Assessment of Captive Environment for Oriental Small-Clawed Otters (*Aonyx cinereus*) in Otter Cafés in Japan

**DOI:** 10.3390/ani14162412

**Published:** 2024-08-20

**Authors:** Nana Ushine, Ayu Kamitaki, Akiyuki Suzuki, Shin-Ichi Hayama

**Affiliations:** 1 One Welfare Education and Research Center, Joint Faculty of Veterinary Medicine, Yamaguchi University, 1677-1 Yoshida, Yamaguchi City 753-0841, Yamaguchi, Japan; 2 Laboratory of Animal Welfare, Department of Animal Health Technology, Yamazaki University of Animal Health Technology, 4-7-2 Minami Osawa, Hachioji City 192-0364, Tokyo, Japan; 3 Wildlife Research Center of Kyoto University, 2-24 Tanaka-Sekiden-Cho, Sakyo 606-8203, Kyoto, Japan; 4 Laboratory of Wildlife Medicine, Department of Veterinary Medicine, Nippon Veterinary and Life Science University, 1-7-1 Kyounan-Cho, Musashino City 180-8602, Tokyo, Japan; hayama@nvlu.ac.jp

**Keywords:** animal café, *Aonyx cinereus*, captive environment, oriental small-clawed otter, proper care

## Abstract

**Simple Summary:**

We evaluated the welfare of oriental small-clawed otters (OSOs; *Aonyx cinereus*) kept in cafés using non-invasive methods. Our assessment focused on the captive environment, utilizing husbandry standards employed in zoos. We surveyed five cafés that agreed to participate from February to April 2023. The results revealed inadequate water facilities in all cafés, with individual housing observed in over half of them. OSO cafés have the feature of disseminating information through customers. Therefore, it is crucial for cafés to rigorously uphold appropriate OSO husbandry conditions. This necessitates incorporating animal welfare concepts into laws regulating such cafés. Thorough management of OSOs in cafés can educate the public about the challenges of OSO ownership in households and potentially mitigate domestic demand for OSOs. Additionally, it is expected to contribute to deterring threats to OSO conservation, such as poaching.

**Abstract:**

This study investigates the captive environments of oriental small-clawed otter (OSO; *Aonyx cinereus*) cafés in Japan, aiming to identify discrepancies with established welfare guidelines. Improved management of these commercial facilities could raise awareness about the difficulties of keeping OSOs as pets and enhance their welfare. Utilizing the role of commercial facilities in public outreach, we consider that the rigorous implementation of proper care practices in these establishments could help mitigate the increasing demand for OSOs domestically and contribute to the conservation of the OSO. In this study, we focused on the critical aspects of the captive environment necessary to maintain the psychological well-being of OSOs and evaluated the captive environments of OSOs housed in animal cafés for OSO welfare using non-invasive methods. Based on zoo husbandry standards, it was found that there were deficiencies in aquatic environments and solitary housing conditions; however, the enrichment tools aligned with the guidelines. The results suggest that deviations from the recommended elements in the environment of OSOs in captivity are associated with a tendency of these OSOs to develop various diseases. It is deemed necessary to amend the regulations governing animal-handling businesses to include welfare criteria, and it is considered essential for each establishment to operate only after ensuring sufficient welfare for the OSOs.

## 1. Introduction

The oriental small-clawed otter (Asian small-clawed otter, *Aonyx cinereus*; OSO) is a carnivorous mammal belonging to the Mustelidae family. They are semi-aquatic mammals that inhabit terrestrial areas adjacent to water bodies, ranging from Southeast Asia to India [1]. In contrast, in captivity, OSOs prefer quiet, dry, and dark places for resting called nest boxes [2]. They are social species, typically residing in groups including mature pairs and their offspring [3]. A few of their aggressive social behaviors include the exclusion of breeding-capable individuals from social groups [4] and the begging behavior of younger individuals [5]. In larger families, potential parental aggression may be observed toward offspring that have reached sexual maturity [4]. The young are weaned between three to four months of age and are simultaneously taught to swim by their parents [6]. After about two years old, they become independent from their parents and start searching for a mate [7]. As a result, they do not form complete herds and instead associate with specific mates. Within a single group, only the parents reproduce; therefore, in environments with limited inter-group interactions, the likelihood of contact between young OSOs decreases.

Three major threats to OSO welfare have emerged in recent years. Of these, habitat degradation is the first threat [8]. Notably, OSOs inhabit wetlands and mangrove swamps in Asia. However, soil erosion into water bodies because of reforestation and deforestation for coffee and tea production and the runoff of pesticides used during reforestation has led to the loss and degradation of the OSO habitats [9]. Further, poaching by humans living in the same area as their habitat for OSO fur [10] and illegal trade by humans not living in the area [11,12,13] are the second and third threats, respectively. As of 2024, OSOs have been classified as a vulnerable species on the International Union for the Conservation of Nature (IUCN) List [8] and have also been included in Appendix 1 of the Convention on International Trade in Endangered Species of Wild Fauna and Flora (CITES), which prohibits all international trade of all such internationally protected species [14].

According to CITES [15], between 2014 and 2020, 83 OSOs exported from Indonesia, one of their native countries, were all imported to Japan, indicating their highest demand in Japan among all countries. According to survey reports from 2000 to 2016, although the number of imports intended for use in zoos has declined in recent years, the number of imports for commercial purposes has remained relatively constant [16].

In Japan, all mammals in the possession of persons are defined as “protected animals”, and protected animals are considered subject to animal abuse under the Act on Welfare and Management of Animals [17]. This law stipulates that individuals who commercially handle and manage protected animals must register all animal-handling businesses with local authorities. Registration requirements include employing a manager to handle the animals. The qualifications for an animal-handling manager are either being a veterinarian or animal nurse or individuals who have studied in an animal-related vocational school and have at least a year of practical experience [17]. Although there is a national-level educational curriculum for specific animal species in veterinarians and animal nurses, no specific educational curriculum exists for the veterinary care and handling of exotic animals [18,19]. Those who have studied in animal-related vocational schools have varying curricula depending on the institution; therefore, the specifics of their knowledge and skills remain unclear. The increase in animal utilization for commercial purposes can be associated with the number of commercial establishments that provide direct animal interaction and observation opportunities to humans. Commercial establishments that provide animals for interaction and observation are referred to as “cafés,” and those with cats (*Felis catus*) are referred to as “cat cafes.” In Japan, the number of cat café establishments has increased from 3 to approximately 300 from 2005 to 2015 [20], and cafés utilizing animals other than cats began to emerge in the second half of the 2000s [21]. However, the exact number of café establishments nationwide and the precise species and individual counts of animals used for commercial purposes remain undisclosed. As café management is expected to continue as an occupation in the country, this may result in a high and sustained demand for acquiring more animals with a lifespan for these establishments, ultimately raising concerns about the potential facilitation of illegal trade, such as poaching.

The evaluation of husbandry conditions—such as diet, cage size, and temperature—is directly related to the Five Domains and the Five Freedoms, which are fundamental to animal welfare assessment. In particular, behavioral interactions with humans are considered critical for assessing animal welfare [22]. Therefore, attention must be given to the interactions between the customers and the otters in such cafés.

Humans often achieve mental well-being through interactions with companion animals [23]. Customers at animal cafés engage with animals as pseudo-owners, enjoying companionship without the responsibilities of actual pet ownership. As a result, they may experience similar mental health benefits as those gained from interactions with their own pets. However, there are no reports on the mental well-being of otters (OSOs) during their interactions with humans in such cafés. An essential factor in ensuring the mental well-being of OSOs is their captive environment. OSOs live in strict family groups, with only specific pairs breeding within each group [24]. During the breeding season, they establish territories to exclude other individuals and sexually mature offspring [25]. Additionally, there have been reports of OSOs in cafés experiencing deaths due to renal failure and accidents such as falls [26]. Considering these ecological characteristics and the reported cases of deaths in such cafés, the captive environments in OSO cafés—being a uniquely Japanese occupational domain—pose a significant threat to OSO welfare [21].

Guidelines for the captive environments for OSOs established by the IUCN/SSC Otter Specialist Group, Otters in the Captivity Task Force, specify appropriate environments based on the behavioral ecology of wild otters [2]. Further, a captive environment is also crucial for the breeding success of OSOs. The successful breeding of otters, including OSOs, depends on the formation of strong bonds between the males and females before mating [27], as well as the design of appropriate habitat environments [28].

To eradicate the inappropriate OSO trade in Japan, improving the appropriate management of domestically bred OSOs, increasing breeding success rates, and distributing domestically bred individuals through facilities, including zoos, are crucial. Therefore, widespread adoption of breeding management, considering the welfare of OSOs, is imperative. Therefore, this study aimed to investigate the captive environments in domestic OSO cafés (hereafter referred to as otter cafés) to conserve OSOs. We investigated the relationship between the applicability of each condition recommended under the captive environment guidelines for otter cafés and the occurrence of diseases related to impairments in both physical and mental well-being.

## 2. Materials and Methods

This study aimed to assess the welfare of OSOs, which are facing concerns about declining populations. Herein, we evaluated the captive environments of OSOs kept in animal cafés using non-invasive methods. Given that OSOs are commercially used in these cafés, invasive investigations could not only compromise their welfare but also potentially disadvantage the café’s operations. Therefore, in this study, we focused on assessing the captive environments of the OSOs with a non-invasive method to evaluate their welfare.

In January 2023, following the methodology outlined by Sigaud et al. [29], we compiled a list of establishments operating otter cafés using a website. Next, between February and April 2023, we conducted field surveys, including both interviews and observations, regarding the captive environments in these cafés. We conducted this survey with the consent of the managers of the OSO cafés under investigation, emphasizing that the study aimed to assess the welfare of OSOs and would not employ invasive methods on the individuals. The survey items were developed with reference to guidelines from the IUCN/SSC Otter Specialist Group, the Otters in Captivity Task Force [2], the New South Wales Department of Industry [30], and the Japanese Association of Zoos and Aquariums [4] (Table 1). Moreover, the survey items were selected with the maximum possible objectivity to avoid implicit subjectivity. For instance, the land area (m^2^) could be verified by comparing the participants’ responses with the measurements recorded by the surveyors. Items related to health management were developed using the formula of Ruiz-Olmo et al. [31] for assessing emaciation in otter species. Free-answer items such as the variety of feeds, materials used for drying off the otters’ bodies, frequency of cleaning the enclosure, and their illness history were quantified using after-coding methods based on keywords in the responses. Based on the survey results, the proportion of the number of types of illness history relative to the otter number (proportion of illness types) was calculated. Subsequently, the number of responses, indicating whether the recommended captive environmental conditions were not met or were unknown (number of non-recommended or unknown conditions) was calculated and compared with the proportion of illness types using Spearman’s rank correlation coefficient in Stata (StataCorp LLC., College Station, TX, USA). Significance was determined using a 95% confidence interval (α = 0.05).

The interviews of otter cafés were conducted through semi-structured interviews [32] with café managers. The surveyors were trained in this method and had previous experience using this method through research activities. To address response bias, we focused on selecting items that could be objectively verified, e.g., number of water areas. To mitigate demand characteristics, the researcher used the deception method. During the consent process, the researcher provided an overview of the study and, upon its conclusion, presented a detailed explanation of the responses and the specifics of the study. The timing of the surveys was decided in advance through consultation with the café managers. The surveys were conducted during the operating hours of these cafés but at times with fewer customers to ensure accuracy and avoid falsification. The survey duration was limited to an hour, which is the basic timeframe allowed for customer visits. Data collection regarding the captive environment involved surveyors visiting the café and obtaining direct responses from the facility managers. This approach was chosen to ensure that the surveyors could measure and observe the captive environment with the manager’s permission if there were unclear responses. Moreover, it was visually confirmed that the OSOs under observation did not exhibit any signs of illness during the survey. The observations were conducted at an appropriate distance to ensure that they did not impact the welfare of the OSOs. This study was conducted in accordance with the Yamazaki University Animal Experiment Ethics Guidelines [33].

## 3. Results

As of January 2023, of the 11 otter cafés in Japan, 5 cafés provided consent for field surveys, and the survey results are summarized in Table 2. Interviews with the café managers were conducted once at each otter café, totaling five interviews. Supplementary information regarding the otters housed in each of these five cafés is provided in Table S1. The average duration of the interviews was 53 min (ranging from a maximum of 90 min to a minimum of 35 min). Among the interviewed cafés, four allowed the surveyors to observe and interact with OSOs, whereas one café only allowed them to observe and not interact with OSOs. The café that did not allow interaction with OSOs cited the reason for denial as the risk of injury because the OSOs were not accustomed to people. However, in all four cafés that allowed interactions with the OSOs, the interaction time ranged from 30 min to 1 h per customer. One person was allowed to interact with one OSO during the interaction. Unless multiple otters were present, the OSO used for the interaction could not escape customer contact during the time set at the café. If an OSO used for the interaction vocalized, bit, or showed aggressive behavior toward customers, however, the interaction was stopped. Outside the interaction time, the OSOs stayed in their assigned cages or pools located away from the customers. All the surveyed cafés managed the OSOs within the premises throughout the day. Two cafés responded that they only moved an OSO when it was unwell or during overall store cleaning. Three out of four otter cafés that housed multiple otters but practiced solitary confinement (Question 2) mentioned aggressive behavior caused by compatibility issues among the otters. One of these cafés also mentioned that interactions with the other otters increased stress. Cafés that kept more than 10 OSOs reported that some of the otters were bred in their cafés (Question 1). The water temperature at the cafés that kept otters outside of the recommended range was between 38 °C and 45 °C (Question 5). Regarding the recommended feed variety (Question 8), four, one, and four cafés provided fish, chicken, and cat feed, respectively. However, one of these cafés provided bread, whereas another provided synthetic animal feed other than cat feed, instead of the recommended otter feeds.

All cafés utilized the recommended materials for drying off the otters’ bodies (Question 12); for example, two, three, and one café provided hemp bags, towels, and a sturdy cushion. As enrichment tools (Question 11), human toys (five cafés), large dog toys (five cafés), stones (two cafés), plastic bottle caps (one café), and glass marbles (one café) were provided. The health management issues included urinary tract issues, such as urinary stones and blood in the urine; digestive issues, including diarrhea; skin issues, including hair loss and wounds; and respiratory issues, including sneezing, runny nose, and eye discharge (Question 18). The proportion of illness types was as follows (number of types of illness history, e.g., urinary, digestive, skin, and respiratory illnesses / number of otters): Café A had 0 (0/2), Café B had 0.14 (3/21), Café C had 0.13 (2/15), Café D had 1 (1/1), and Café E had 1.5 (3/2). The number of non-recommended or unknown conditions were as follows: Cafés A, B, C, D, and E had six, four, three, seven, and seven such conditions, respectively. When examining the correlation between the number of items not meeting welfare standards and the proportion of illness types, no significant correlation was found; however, a positive correlation was observed (*r* = 0.62, *p* = 0.27).

## 4. Discussion

This study revealed the captive environment of commercial OSO facilities. Among the 11 establishments confirmed through web research, field surveys covered fewer than half. Consequently, the actual state of the otter cafés nationwide remains unclear. To ensure proper care of the otters, it is necessary to reconsider the appropriate survey methods to understand the captive environments in all these cafés. In OSO cafés, interactions involve both physical contact with customers and observations. No consideration of animal welfare was there while allocating the time for physical contact, and both types of interactions were aimed at business purposes. Considering the cases of stress-induced deaths and fall-related deaths attributed to these interactions [26], OSOs may experience a decline in their mental well-being because of the interactions set by the cafés. The captive environment in OSO cafés is crucial for maintaining the physical health and mental well-being of OSOs. Additionally, it is necessary to provide hiding places and secure resting areas for OSOs to prevent constant observation during café hours.

Notably, the water area provided in all the cafés was smaller than the recommended level. However, as breeding success has been confirmed in cafés managing more than 10 otters, the scale of water recommended by zoos may not be an essential environmental factor when evaluating the welfare of OSOs related to reproductive behavior. The enrichment corresponded to the guidelines in all cafés. Unlike zoos, otter cafés operate in commercial buildings or eateries, posing a challenge to providing sufficient space for OSOs. A relationship was observed between the proportion of illness types and the number of non-recommended or unknown conditions. As interview responses regarding illness history depend on the café managers’ observation and responsibility toward OSOs, the café that reported no illness history may have provided an underestimated response. Considering the survey results obtained from all cafés, it was suggested that a high number of non-recommended or unknown conditions correlated with the tendency of captive OSOs to develop various diseases. Therefore, meeting the standards of captive environmental conditions that ensure OSO welfare, such as the guidelines used in this study, is critical for improving OSO welfare. Additionally, to further suppress domestic demands, it is necessary to provide optimal captive environments in otter cafés that meet all the guideline conditions. This initiative will raise awareness regarding the difficulty of maintaining OSOs in ordinary households and may enhance the welfare of OSOs in otter cafés.

Based on the social structure of OSOs, the management of OSOs as family units is recommended for effectively managing OSOs in commercial facilities [3]. Managed OSOs require group housing with constant monitoring for compatibility within the limited available space. In commercial facilities housing multiple individuals, a few otters were isolated from others as they were considered aggressive toward others and were stressed by interactions with other otters. Aggressive behaviors in OSOs can arise because of certain ecological factors, such as social group dynamics and reproduction. Therefore, managers should understand both the aggression and ecological actions of OSOs and consider providing suitable environments and group compositions. Instead of remaining confined to their preferred resting conditions during active periods, some otters may exhibit an increased variety of behaviors as a result of their interactions with visitors [34]. Individual differences and daily activity rhythms are factors related to behavioral expressions [35], so managers must understand their individual activity rhythms and provide the appropriate environments based on detailed observations.

Considering the habitats of OSOs in the wild, the land areas must be larger than the water areas. In addition, providing materials for drying off their bodies is crucial for otter welfare because incomplete drying may cause dermatitis [36]. The diseases associated with captive OSOs can occur throughout the body. Okamoto et al. [26], based on the medical records of captive OSOs in Japan between 2010 and 2019, reported that the most common illness in captive OSOs was renal calculi, followed by pneumonia and dehydration, which were attributed to the captive environment and feed. Although no emaciation was observed in the surveyed otter cafés, more than half reported health issues in OSOs. As otters are potential carriers of zoonotic diseases, providing an environment that enhances their well-being may contribute to preventing disease transmission to humans. In fact, visceral pentastomiasis [37] and bovine tuberculosis [38] have been reported in OSOs. Additionally, otters can cause wound infections in humans, including tetanus, because of their sharp teeth and robust jaws for preying on crabs and shellfish [5]. If they are not properly quarantined, their handling also carries the risk of rabies [39]. Therefore, managers should acquire knowledge of disease management and prioritize the well-being of otters over providing customer entertainment. Further, in addition to proper introduction and management, managers must thoroughly explain the risks of injuries and infections to customers before offering the otters.

CITES Appendices 1 and 2 entail different regulations for animal handling. Under Appendix 1, imports and exports for purposes other than scientific research are prohibited. Additionally, domestic trade is regulated by the Act on Conservation of Endangered Species of Wild Fauna and Flora, which imposes registration requirements for imports and exports, prohibits display and advertising for sale, and prohibits transfers [40]. Conversely, under Appendix 2, commercial transactions are permissible, and exemptions from the Act on Conservation of Endangered Species of Wild Fauna and Flora allow for display and transfer for sale. As individual registration is not required, distinguishing between wild-caught and captive-bred otters is difficult once they enter domestic circulation in the market [41]. However, OSOs were listed in Appendix 1 of CITES in 2019 because of their illegal trade in Japan. Commercial facilities help increase public outreach and awareness and are often highlighted in the media and social networking services. By ensuring proper care of captive otters, commercial facilities can contribute to raising awareness of animal welfare and deterring the increased demand for OSOs. Therefore, it is necessary to incorporate animal welfare criteria into the registration standards for animal handling businesses under the Act on Welfare and Management of Animals. Through this legislative amendment, café owners would be able to commence commercial handling of OSOs only after ensuring adequate welfare environments for them. Additionally, the exposure of visiting customers to the optimal captive environments of otters can help raise awareness among individuals desiring to privately own OSOs, highlighting the challenges of keeping them in ordinary households.

## 5. Conclusions

This study investigated whether otter cafés maintain the appropriate captive environments conducive to the welfare of small-clawed otters using non-invasive methods. Based on zoo husbandry standards, it was found that there is a deficiency in aquatic environments. From the relationship between the conditions of the captive environment and the illness history of the OSOs, it was suggested that a higher number of non-recommended or unknown conditions correlates with a tendency to develop various diseases. As a result of this investigation, it is deemed necessary to amend the regulations governing animal-handling businesses to include welfare criteria, and it is considered essential for each establishment to operate only after ensuring sufficient welfare for the small-clawed otters.

## Figures and Tables

**Table 1:** The field survey items.

No.	Category	Questions	Recommended Environment Specifications
1	Total number of animals kept, and rooms or cages	Total number of animals being kept	Unspecified
2		Total number of animals exhibited in one room or cage	Keeping multiple animals [2]
3	Land and water area	Water area per animal	2.5m^2^ or more [30]
4		Water depth	1.0 m or more [30]
5		Water temperature	18.3 to 29.4 °C [2,4]
6		Land temperature	22.2 to 24.4 °C [2,4]
7		Ratio of land to water area	Land area is more than twice water area [2,4]
8	Feeding	Variety	Chick, rabbit with skin, lean meats, cat food, grated fruits and vegetables, fish, as well as vitamin supplements, mealworms, snails, earthworms, crickets, crayfish, shellfish, raisins, and unsalted peanuts as supplementary feed [2]
9		Quantity	About 20% of body weight [2]
10	Enrichment	Enrichment tool	providing them [2]
11		Features of the tool	Durable and environmentally friendly [2]
12		Material for drying off their bodies	Towels, hay, rice straw, plants for landscaping, fallen leaves [4]
13		Frequency of cleaning the enclosure	The daily partial cleaning rotation [2,4]
14	Health management	Body weight (kg)	Unspecified
15		Non-emaciated physical condition (K)	1.0 or more [31]
16		Microchip implantation	Unspecified
17		History of illness	Unspecified
18		Details of illness	Unspecified

**Table 2:** Results of the field survey.

No.	Category	Questions	Results
1	Total number of animals kept, and rooms or cages	Total number of animals being kept	One animal: 1
			More than two but less than ten: 2
			Ten or more: 2
2		Total number of animals exhibited in one room or cage	One animal: 4
			Two or more: 5
3	Land and water area	Water area per animal	Less than 2.5m^2^: 5
			2.5m^2^ or more: 0
4		Water depth	Less than 1.0 m: 4
			1.0 m or more: 1
5		Water temperature	Recommended range: 1
			Outside the recommended range: 2
			Not responding: 2
6		Land temperature	Recommended temperature range: 2
			Outside the recommended range: 3
7		Ratio of land to water area	Less than twice: 3
			More than twice: 2
8	Feeding	Variety	Recommended feed: 10
			Unrecommended feed: 2
9		Quantity	Less than 20%: 2
			About 20% to 25%: 1
			Not determined: 2
10	Enrichment	Enrichment tool	Providing them: 5
			Not providing them: 0
11		Features of the tool	Possessing features: 5
			Not possessing features: 0
12		Material for drying off their bodies	Recommended material: 5
			Unrecommended material: 0
13		Frequency of cleaning the enclosure	Recommended cleaning: 5
			Unrecommended cleaning: 0
14	Health management	Body weight (kg)	3.0–5.0 (mixed sexes; average value 3.68)
15		Non-emaciated physical condition (K)	Male: 1.14–2.11 (median value 1.19)
			Female: 1.19–1.25 (median value 1.22)
16		Microchip implantation	Implanted: 3
			Not implanted: 2
17		History of illness	Yes: 4
			No: 1
18		Details of illness	Urinary: 3
			Digestive: 1
			Skin: 3
			Respiratory: 2

## Data Availability

The data presented in this study are available from the corresponding author upon reasonable request.

## Supplementary Materials

The following supporting information can be downloaded at: https://www.mdpi.com/article/10.3390/ani14162412/s1, Table S1: Supplementary information regarding the otter cafés and oriental small-clawed otters (OSOs) housed in each of the five cafés.
