# Supplementary material for: Assessment of Captive Environment for Oriental Small-Clawed Otters (Aonyx cinereus) in Otter Cafés in Japan
Source: Animals (Basel). 2024 Aug 20;14(16):2412. doi: 10.3390/ani14162412 (PMC11350695; doi:10.3390/ani14162412)
Supplement: Supplementary file 1 [file animals-14-02412-s001.zip › animals-3141855-supplementary.pdf]

Table S1. Supplementary information regarding the otter cafés and oriental small-clawed otters (OSOs) housed in each of the five cafés.

Category	Results in numbers
Origin	Wild-caught: 0 (three cafés); 3 (one café)<br>Captive bred: 1 (one café); 2 (one café); 15 (one café); 19 (four cafés)<br>Unknown: 2 (one café)
Sex of OSOs in each store	1 male (Café A); 2 males (Café B); 1 male and 1 female (Café C);<br>6 males and 9 females (Café D); 11 males and 9 females (Café E)
Years of café operation	7 (one café); 6 (two cafés); 5 (one café); 4 (one café)
Cohabiting animal species	Lop-eared rabbit (*Oryctolagus cuniculus*) (one café); Four-toed hedgehog (*Atelerix albiventris*) (two cafés); Chinchilla (*Chinchilla lanigera*) (one café); Bengal cat (one café); Nyasa lovebird (*Agapornis lilianae*) (one café); Sugar glider (*Petaurus breviceps*) (two cafés)
Hygiene procedures recommended by each store for customers	Hand sanitizer with 70% ethanol to be used upon entering and exiting the café (all cafés); Strictly no shoes allowed (one café)
